# Emerging Opportunities to Study Mobile Element Insertions and Their Source Elements in an Expanding Universe of Sequenced Human Genomes

**DOI:** 10.3390/genes14101923

**Published:** 2023-10-10

**Authors:** Scott E. Devine

**Affiliations:** Institute for Genome Sciences, Department of Medicine, and Greenebaum Comprehensive Cancer Center, University of Maryland School of Medicine, Baltimore, MD 21201, USA; sdevine@som.umaryland.edu; Tel.: +1-(410)-706-2343

**Keywords:** *Alu*, LINE-1, L1, SVA, mobile element insertions, human genomes

## Abstract

Three mobile element classes, namely *Alu*, LINE-1 (L1), and SVA elements, remain actively mobile in human genomes and continue to produce new mobile element insertions (MEIs). Historically, MEIs have been discovered and studied using several methods, including: (1) Southern blots, (2) PCR (including PCR display), and (3) the detection of MEI copies from young subfamilies. We are now entering a new phase of MEI discovery where these methods are being replaced by whole genome sequencing and bioinformatics analysis to discover novel MEIs. We expect that the universe of sequenced human genomes will continue to expand rapidly over the next several years, both with short-read and long-read technologies. These resources will provide unprecedented opportunities to discover MEIs and study their impact on human traits and diseases. They also will allow the MEI community to discover and study the source elements that produce these new MEIs, which will facilitate our ability to study source element regulation in various tissue contexts and disease states. This, in turn, will allow us to better understand MEI mutagenesis in humans and the impact of this mutagenesis on human biology.

## 1. Introduction

**Figure 1:**
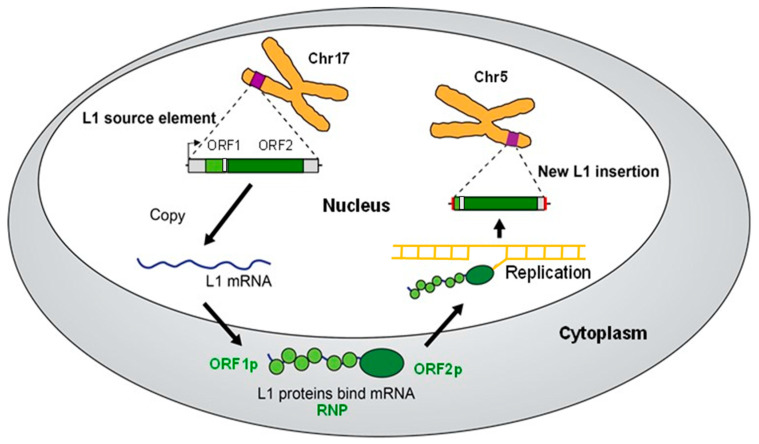
L1 retrotransposition cycle. The L1 retrotransposition cycle that produces a new L1 insertion (MEI) is depicted. Full-length L1 source elements with two intact ORFs encode potentially active ORF1p and ORF2p proteins (upper left—ORF1 in light green, ORF2 in dark green). In this case, the L1 source element is located on chromosome 17 (Chr17). The source element is transcribed from the internal L1 promoter (arrow) to generate L1 mRNA (blue). The L1 mRNA is exported to the cytoplasm, where the ORF1 and ORF2 regions are translated to produce ORF1p and ORF2p. These proteins bind to the mRNA that generated them through a process called cis-preference to generate an L1 RNP. The RNP is imported back into the nucleus, where the process of target-primed reverse transcription (TPRT) uses the mRNA template to generate an L1 MEI at a new genomic location. In this case, the new insertion is located on chromosome 5 (Chr5). A double-stranded L1 MEI likely is generated by similar steps as the first strand. Note that new insertions frequently are 5′ truncated (as depicted) and are flanked by new target site duplications (red). *Alu* and SVA use a similar process by substituting their RNAs and hijacking the L1 machinery.

Mobile genetic elements occupy approximately half of the human genome [1]. However, only three element classes, i.e., *Alu*, LINE-1 (L1), and SVA elements, remain actively mobile and continue to mutagenize human genomes today [2,3,4,5,6,7,8,9,10,11,12,13,14,15]. All three of these element classes are non-LTR retrotransposons that are mobilized through RNA intermediates using the protein machinery that is encoded by the L1 retrotransposon. Specifically, the L1-encoded proteins ORF1p and ORF2p generate new *Alu*, L1, and SVA “offspring” mobile element insertions (MEIs) through a mechanism that is termed target primed reverse transcription (TPRT) [16] (Figure 1). ORF1p encodes a nucleic acid chaperone [17,18], whereas ORF2p encodes an endonuclease (EN) [17,19] and a reverse transcriptase (RT) [17,20]. Since the L1 machinery mobilizes all three of these element classes [17,21,22,23], new *Alu*, L1, and SVA MEIs share characteristic features of L1 elements, including L1-like target site duplications (TSDs), poly (A) tails, and interior mutations that may be created by the error-prone L1 reverse transcriptase.

New MEIs are generated both in the germline [6,7,8,9,10,11,12,13,14,15] and in at least some somatic human tissues (i.e., epithelial cancers [6,24,25,26,27,28,29,30], reviewed in [31,32] and neuronal tissues [33,34,35,36,37,38], reviewed in [39]). Germline MEIs have been implicated in several dozen human diseases, including hemophilia [40], neurofibromatosis [41], and Duchenne muscular dystrophy [42] (reviewed in [43,44]). Somatic MEIs have been implicated in a wide range of epithelial cancers, including colon, lung, liver, esophageal, and pancreatic cancer (reviewed in [31,32]) and in several neurological diseases, including Rett Syndrome, Aicardi-Goutieres Syndrome, Schizophrenia, Amyotrophic Lateral Sclerosis (ALS), and normal aging (reviewed in [39]). MEIs typically cause diseases by disrupting gene function through insertional mutagenesis of exons or other functionally important sequences. Therefore, both germline and somatic MEIs should be fully discovered, along with other forms of human genome variation, in studies involving population genetics, human diseases, and clinical genomics.

We are now entering a new era of MEI discovery where whole genome sequencing (WGS) and bioinformatics analysis are becoming the dominant methods to identify and study MEIs [12,13,14,15,45,46,47]. As the cost of Illumina WGS continues to drop, the universe of WGS “Big Data” that is available is rapidly expanding, with some studies performing WGS on 100,000 or more human genomes. Likewise, as the cost of PacBio and other long-read sequencing becomes more affordable and accurate, the number of telomere-to-telomere human genome assemblies is rapidly expanding through the work of the Human Genome Structural Variation Consortium (HGSVC) [47], the Human Pangenome Reference Consortium (HPRC) [48], the Telomere-to-Telomere (T2T) [49] project, and the All of Us project (https://allofus.nih.gov/, accessed on 29 September 2023). This revolution in “Big Data” production is now presenting unprecedented opportunities and challenges to study the impact of MEIs on human genomes, phenotypes, and diseases. In this review, I examine the transition that has begun to occur from historical studies that initially established a role for MEIs in both germline and somatic human diseases to the WGS-based approaches that will facilitate this new revolution in human MEI discovery and analysis. I explore the opportunities that are emerging using WGS sequencing data to study MEIs in humans and the challenges that we face if we wish to study the impact of these new MEIs on human biology and diseases.

## 2. The Transition from Pre-Genome MEI-Discovery to WGS

The earliest studies that implicated human MEIs in human diseases were published in the late 1980s and early 1990s, well before the human genome had been sequenced. The earliest study was published by Kazazian and colleagues in 1988, where they reported two independent germline L1 insertions that disrupted the 14th coding exon of the *Factor XIII* gene in patients with hemophilia A [40]. Both of these disease-causing MEIs were considered to be de novo insertions, as neither was detected in the parents of the patients [40]. In 1991, Francis Collins and colleagues discovered a germline *Alu* insertion that disrupted the *NF1* gene in a patient with neurofibromatosis, demonstrating that germline *Alu* insertions also can cause diseases [41]. In a third milestone study that was published in 1992, Miki et al. discovered a somatic L1 insertion that disrupted the 16th coding exon of the *APC* tumor suppressor gene in a patient with colorectal cancer (CRC) [24]. The L1 insertion was found in the tumor but was absent from the adjacent normal tissues, indicating that it must have been mobilized in somatic colorectal tissues. Finally, SVA elements also can cause diseases when they are mobilized in the germline (e.g., see reference [50]). Overall, these studies collectively indicate that L1 elements are actively mobile in both germline and somatic human tissues, whereas *Alu* and SVA elements are active mostly in the germline. Moreover, all three of these elements can cause diseases when newly-mobilized copies disrupt genes.

In many regards, these initial studies were very insightful in terms of what would follow historically. Several dozen disease-causing *Alu*, L1, and SVA MEIs subsequently have been identified in both germline and somatic tissues during the ~35 years that have elapsed since these initial studies (reviewed in: [31,32,43,44]). Some of these studies were performed in the pre-genomic era using methods that were somewhat laborious and time-consuming. For example, the earliest study outlined above in hemophilia [40] used Southern blot hybridization to discover the disease-causing MEIs, a method that has become largely obsolete today. These early studies also were limited to a small subset of well-characterized genes that had been cloned and sequenced with library-based approaches. After these initial studies, PCR-based approaches were used to discover and study polymorphic MEI copies throughout the human genome, including those that caused diseases (e.g., [31,32,43,44,51,52,53]). Broader methods such as MEI display and methods involving genome-wide amplification and sequencing of young MEI subfamilies also have been very effective for discovering polymorphic MEIs over the past 10–15 years (e.g., [6,9,10,33]). Nevertheless, such methods are rapidly being superseded by WGS, which is ushering in a new era of MEI discovery on unprecedented scales in humans. Since the WGS data frequently have been produced by existing projects such as the 1000 Genomes Project, TOPMed, or several long-read consortium projects, there are often no sequencing costs associated with the WGS discovery approach, and the only challenge is to obtain the sequences and mine the MEIs from the WGS data. Thus, WGS-mediated MEI discovery arguably will become the dominant approach for studying MEIs over the next several years and will provide a quantum leap in our understanding of MEI mutagenesis in thousands, if not millions, of humans.

## 3. The 1000 Genomes Project: MEI Discovery on a Population-Scale Using WGS Data

The 1000 Genomes Project has led the way in developing new MEI discovery and analysis tools that could be applied to large WGS data sets. Starting with the 1000 Genomes pilot project of 185 genomes, Stewart et al. developed a novel computational approach that exploited Illumina paired end and split read data to discover 5371 non-reference (non-REF) *Alu*, L1, and SVA MEIs (4500 *Alu*, 792 L1, and 79 SVA MEIs [11]). Additional tools were developed during the later phases of the 1000 Genomes Project (phases 1, 2, and 3), including the Mobile Element Locator Tool (MELT), Retro-seq, and Tangram [12,13,14,15,54,55]. MELT was used to generate the final call sets for the project, leading to the discovery of 22,723 non-REF MEIs in 2504 genomes (17,543 *Alu*, 4118 L1, 1062 SVA [14]). Initial studies with the 1000 Genomes Project samples were performed with relatively low coverage Illumina WGS data (~7× average coverage [12,13,14]). More recently, MEI discovery has been performed with high coverage (30–40×) Illumina WGS data in 3202 samples from the 1000 Genomes Project, including 602 additional trio genomes (a child and two parents [15,56]). 54,537 MELT calls were generated with these high-coverage genomes, including 31,814 additional MEIs compared to the low-coverage studies (largely due to the increased coverage and additional genomes that were analyzed) [15,56].

What did we learn from these population-scale MEI discovery studies using WGS data generated by the 1000 Genomes Project? First, from the 7× genomes, we learned that the average human harbors an average of 1093 polymorphic non-REF MEIs and that this average varies from 1007 to 1220 in the five major continental populations that were studied by the project (African, American, East Asian, European, and South Asian; abbreviated AFR, AMR, EAS, EUR, and SAS, respectively) [12,13,14]. AFR individuals had the highest average number of non-REF MEIs (1220), which is consistent with the greater diversity of AFR populations; individuals in the remaining populations had lower averages (AMR = 1007; EAS = 1085; EUR = 1095; SAS = 1056) [13]. The number of MEIs per individual in higher coverage genomes (30×) was, as expected, higher; however, the same trends were observed in terms of the relative numbers of MEIs per individual in the five superpopulations (15). Most of the non-REF MEIs discovered in these studies were relatively rare (i.e., had minor allelic frequencies or MAFs below 1%) and were underrepresented in functionally important regions of genes, indicating that new MEIs in such regions often are detrimental [12,14,15]. We also observed diverse patterns of MEI locus sharing across the five major continental populations and the 26 diverse subpopulations that were studied [12,14,15]. This includes polymorphic non-REF MEI loci that were (1) shared by all humans, (2) shared by a subset of populations, and (3) population-specific. These diverse sharing patterns likely were caused by many factors, including the diversity of MEI generation in populations, as well as differences in inheritance, admixture, positive and negative selection, and the introgression of MEIs from Neanderthal/Denisovans into modern humans [12,14,15].

We also found that the very same subfamilies of *Alu*, L1, and SVA were active in ancient hominids that are active in modern humans [14]. The Out of Africa (OOA) model of human demographic history was confirmed with phylogenetic trees and PCA analysis using homoplasy-free MEIs as markers [12]. Homoplasy-free Ancestry Informative Markers (AIMs) likewise were identified that could potentially be used to track the ancestry of individuals from specific populations [12]. We also noted that the genomic distributions of non-REF *Alu*, L1, and SVA MEIs are fairly random, although some constraints were noted that were imposed by fluctuations in GC content in the human genome. Some areas of the genome were inaccessible to Illumina WGS (there were no MEI measurements in these regions); however, these regions are likely to become more accessible with T2T long-read genome assemblies from the HGSVC, HPRC, T2T, and All of Us projects [47,48,49]. Finally, many full-length Human-specific L1 (FL-L1Hs) and SVA source elements are active in the 1000 Genomes populations [14,15,47]. Thus, the 1000 Genomes Project has been a rich resource to discover and study human MEIs.

## 4. Emerging Opportunities to Discover MEIs Using Population-Scale WGS

Illumina and long-read WGS are rapidly becoming the main tools of human genetics, and as the costs of WGS continue to drop, the universe of WGS data that will be available for MEI discovery will continue to expand over the next several years. Likewise, many of these studies will be focused on disease cohorts involving thousands of patients with a given trait or disease. For example, the TOPMed project is performing WGS in cohorts of patients with specific traits and diseases related to heart, lung, blood, and sleep physiology. Recently, we used MELT to examine 1112 Amish and 3331 Jackson Heart Study individuals from the TOPMed project using the 30× Illumina WGS data that were generated by the project [15]. The TOPMed study is expected to generate at least 300,000 Illumina whole genome sequences at 30× coverage, which will provide additional opportunities to study the impact of MEIs on specific traits and diseases.

As outlined above, the HGSVC [47], HPRC [48], and T2T [49] projects collectively are generating hundreds of assembled long-read genomes that are sorted into two haplotypes for each chromosome. In addition to PacBio HiFi long reads, some of these projects also are using Oxford Nanopore long reads as scaffolds to assemble these genomes [48,49] along with a variety of other technologies such as Bionano optical maps, single-cell DNA template strand sequencing (Strand-seq), and high-coverage Hi-C Illumina short-read sequencing [48,49]. These hybrid approaches are providing highly accurate and more complete human genome sequences that span more of the repetitive regions compared to short-read Illumina sequencing. Likewise, the All-of-Us project is expected to sequence at least one million human genomes with PacBio long-reads over the next few years (https://allofus.nih.gov/, accessed on 29 September 2023). When combined with large-scale Illumina projects such as TOPMed and the many smaller Illumina projects that involve a few hundred or a few thousand samples that are available from dbGaP (https://www.ncbi.nlm.nih.gov/gap/, accessed on 29 September 2023), we can expect that the aggregate number of sequenced genomes that focus on understanding the genetic basis of human traits and diseases will grow to millions over the next decade. Collectively, this will represent a rich resource to study the impact of MEIs on human traits and diseases.

Several population-scale studies also have been launched to study human cancers, such as the Cancer Genome Atlas (TCGA) and the Pan-Cancer Analysis of Whole Genomes Consortium (PCAWGC) project (https://www.cancer.gov/ccg/research/genome-sequencing/tcga, accessed on 29 September 2023). The WGS data structures for these somatic studies are slightly different from those of germline studies, as they provide WGS data from normal/tumor tissue pairs. Somatic MEIs are identified by comparing the MEIs that are found only in the tumors with those discovered in both the tumor and adjacent normal tissues (e.g., [25,26,27,28,29,30,31,32]). Somatic MEIs also are being studied in the brain, where such insertions frequently are generated [33,34,35,36,37,38,39]. Finally, the newly launched NIH Common Fund SMaHT project will explore somatic MEIs in many additional normal human tissues (https://commonfund.nih.gov/smaht, accessed on 29 September 2023).

## 5. Challenges Associated with Scaling up MEI Discovery to Meet the Demands of These Data-Intensive Projects

There are two major hurdles as we enter this new era of MEI discovery in WGS data: (1) the scalability of MEI discovery algorithms, and (2) the availability or portability of WGS data. The 1000 Genomes Project was initially one of the largest WGS-based MEI discovery projects that was attempted, with 2504 (and later, 3202) WGS samples, and this proved to be quite challenging in many regards. For example, some of the algorithms that were used both within the 1000 Genomes Project and outside of it would be expected to require almost a year of runtime to perform MEI discovery in the WGS samples that were generated for the 1000 Genomes Project (particularly for the high coverage genomes) [14,15]. Clearly, this would not be practical in the context of large Illumina WGS-based studies, particularly as projects begin to tackle tens (and even hundreds) of thousands of WGS samples. MELT was engineered to meet the demands of such studies, as it was developed within the context of the 1000 Genomes Project to face the demands of MEI discovery on these scales. As we tackled high coverage (30×) 1000 Genomes Project samples, we found that earlier versions of MELT were relatively slow with the higher coverage samples, and it took months to process a few thousand genomes on our local grid (compared to ~three weeks for 2504 low coverage samples). This forced us to re-examine the scalability of MELT with high coverage Illumina genomes, and we increased the efficiency of MELT by improving the code and by implementing MELT in the cloud (15). We then processed the 3202 high-coverage Illumina genomes in ~two weeks (instead of several months on our local grid). These analyses were further facilitated by accessing the genome sequences in the cloud rather than by downloading and processing them locally [15].

However, as the coverage and number of samples continue to grow, there is an incentive to further improve the code of MEI discovery tools to bring down the costs of running such tools, particularly in the cloud. For very large projects such as TOPMed, the analysis of 100,000 genomes at $5 per sample would be $500,000. In contrast, at $1 per sample, these costs would be reduced to $100,000 through further improvements in scalability and efficiency (a significant savings). If the cost per sample can be reduced to $1 or less, the costs associated with doing smaller, more focused studies in individual labs would be well within the budgets of most funded labs. Therefore, improving the efficiency and scalability of MEI discovery algorithms remains an important area of research.

## 6. Full-Length L1 Human-Specific (FL-L1Hs) Source Elements

FL-L1Hs source elements are the only autonomous transposons in humans. Such elements are 6 kb in length, and as outlined above, they encode the mobilization machinery that is necessary not only for L1 retrotransposition but also for *Alu* and SVA retrotransposition. Only a relatively small subset of L1 elements is capable of retrotransposition for several reasons. First, at least 73.5% of L1 elements in the human genome are 5′-truncated, which generally renders them inactive [15]. Many FL-L1Hs elements have interior mutations that disrupt promoters, ORF1, and/or ORF2, and such elements often are inactive as well. Elements that cannot be expressed (perhaps due to unfavorable genomic locations) likewise cannot serve as active source elements. Thus, a major challenge moving forward is to identify and study the FL-L1Hs source elements that can drive the retrotransposition of L1, *Alu,* and SVA insertions in the germline and somatic tissues vs. those that cannot.

Some of the earliest studies of FL-L1Hs source elements were motivated by determining whether L1 is a human transposable element, as several early clues seemed to indicate. For example, Adams et al. identified a moderately repetitive element in the human genome that is ~6.4 Kb in length, and they suggested that this element might represent a transposon [57]. The element studied by Adams et al. is equivalent to the *Kpn* I family of relatively large repeats, which was studied by several labs in humans and monkeys. These *Kpn* I studies turned out to be some of the earliest studies examining LINE-1 or L1 elements, as *Kpn* I repeats are equivalent to LINE-1 elements. Shortly thereafter, the Singer lab identified a ~6.5 Kb cytoplasmic RNA transcript in NTera2D1 cells that was likely a transposition intermediate of *Kpn* I/LINE-1 elements [58]. Scott et al. developed a consensus LINE-1 element sequence from available LINE-1 sequences (including the original 6.4 repeat that was found by Adams et al. downstream of the β globin gene) [59]. The Scott et al. consensus is 6 kb in length and potentially encodes two open reading frames (ORF1 and ORF2), where the predicted ORF2 protein has homology to reverse transcriptases [59].

After these initial studies, the pursuit of FL-L1Hs elements was largely driven by a desire to understand the source elements that produced some of the earliest disease-causing L1 insertions. For example, following the landmark study of Kazazian et al. with the *Factor VIII* gene in patients with hemophilia A, the Kazazian group identified a source element on Chr 22 that was the likely progenitor of the de novo L1 insertion that disrupted the *Factor VIII* gene in patient JH27 [60]. This progenitor candidate (L1.2B) was identified using a 20-mer oligonucleotide that had three unique sequence mutations compared to the Scott et al. consensus [59,60]. The sequenced FL-L1Hs (L1.2B) element had two intact ORFs and was identical in sequence to the L1 offspring insertion that disrupted the *Factor VIII* gene in patient JH27. Although the Scott et al. consensus predicted two ORFs, none of the L1 sequences that were used to construct that consensus had two intact ORFs, and the L1.2B element was the first FL-L1Hs source element copy that was discovered with two intact ORFs [60]. Later, two alleles of the L1.2 source element (L1.2A and L1.2B) were shown to be active in a cell-culture-based L1 retrotransposition assay [17,61]. Many additional functional FL-L1Hs source elements have been identified using either interior mutations that are identical between source and offspring elements or by tracking source/offspring relationships using 3′ transductions [14,15,28,29,60,62,63,64,65,66,67].

## 7. Large-Scale Studies of FL-L1Hs Source Elements in Human Genomes

A handful of studies now have discovered and examined over 1000 FL-L1Hs elements in human genomes. Kazazian and colleagues examined the BAC clones that had been sequenced by the human genome project and identified 90 FL-L1Hs reference (REF) elements that had two intact ORFs in the December 2001 “freeze” of the draft human genome sequence [53]. They tested 82 of these FL-L1Hs elements in a cell-culture-based assay for retrotransposition and found that eight of the elements were highly active “hot L1” source elements [17,53]. Beck et al. later sequenced 68 non-REF FL-L1Hs elements that were identified in cosmid clones from eight diverse humans and tested 67 of these elements in the cell-culture assay for retrotransposition [7,17]. They found that 37/67 (55%) of these elements were highly active in the cell culture assay [7] (compared to 8/82, or 9.8%, of the REF elements tested in the Brouha et al. study [53]). These data indicate that the non-REF collection of FL-L1Hs elements studied by Beck et al. was more enriched for younger, hot L1′s [7] compared to the REF elements studied by Kazazian and colleagues [53].

Our lab recently identified 3728 FL-L1Hs elements from five WGS and whole exome sequencing projects using MELT and CloudMELT [15]. We found that the number of non-REF FL-L1Hs elements varied considerably between diverse human populations and across individuals within these populations. For example, individuals within the 1000 Genomes AFR population had more non-REF FL-L1Hs copies (Average = 48.1/individual) than individuals in the remaining super populations (Averages: SAS = 45.0; AMR = 43.4; EUR = 43.2; EAS = 42.9). Moreover, although the number of non-REF FL-L1Hs elements in all 1000 Genomes individuals averaged 44.3, this number varied from 25 to 63 [15]. On the one hand, having fewer FL-L1Hs elements (i.e., 25) might be considered an advantage since we might expect lower levels of MEI mutagenesis compared to having 63 FL-L1Hs elements. However, if all of the 25 elements are highly active “hot L1′s” that are highly expressed and all 63 are non-hot L1′s that are tightly repressed, MEI mutagenesis might be much higher in the individual with 25 non-REF FL-L1Hs elements. Since most non-REF FL-L1Hs elements in these individuals are young and belong to the most active L1 subfamilies [7,15], having fewer non-REF FL-L1Hs elements might be expected to produce lower levels of MEI mutagenesis. Nevertheless, more work is necessary to measure the mutagenic threat that is posed by non-REF FL-L1Hs elements across diverse individuals in germline and somatic tissues.

We followed up these studies with long PCR to amplify 698 of these FL-L1Hs elements and sequenced them with PacBio long reads [15]. The majority of these elements (519/698, or 74.4%) had two intact ORFs and belonged to the youngest and most active L1-Ta1d subfamily [15]. Thus, many of these elements would be expected to be capable of retrotransposition. We also identified three new subfamilies of FL-L1Hs elements within the L1-Ta1d subfamily that represent the most active subfamilies identified to date. A large number of interior mutations were identified in these 698 sequence-resolved FL-L1Hs elements, including mutations that eliminated CpGs in the L1 promoter along with synonymous and non-synonymous codon changes within ORF1 and ORF2 [15]. A major challenge moving forward will be to determine more fully which of these FL-L1Hs elements are active in the germline, somatic tissues, or cultured cells, and to identify elements that continue to mutagenize human genomes.

The HGSVC recently published a collection of 637 sequence-resolved FL-L1Hs elements that were discovered from PacBio long-read WGS assemblies [47]. As outlined in the studies above, the majority of these young, non-REF FL-L1Hs elements (393/637, or 61.7%) had two intact ORFs and, thus, could potentially be active. An important aspect of long-read assembly approaches (Pac and others) is that they provide the full interior sequences of the MEIs [47], whereas short-read approaches provide only the sequences around the insertion junctions [12,13,14,15,54,55,68,69]. Short-read studies require a follow-up step to sequence the full interiors of MEIs [15], whereas long-read assembly approaches do not [47,70,71]. It is already clear from the assembled PacBio genomes that have been generated by the HGSVC that these new approaches will revolutionize our understanding of MEIs (including FL-L1Hs source elements) [47]. These long-read assemblies provide information on FL-L1Hs genomic locations, ORF status, and interior mutations that allow us to identify elements that arose from the youngest L1 subfamilies. Together with the FL-L1Hs projects outlined above, these fully sequenced FL-L1Hs elements will provide a resource for future studies to examine the activities of these elements and their regulation in various tissues.

These long-read approaches also are providing access to additional genomic compartments that were not accessible with Illumina short-read technologies, leading to increased MEI discovery [47]. Assembled PacBio genomes already are recovering MEIs in previously inaccessible genomic compartments, and we expect this to expand into centromeres, telomeres, segmental duplications, and other repetitive regions, particularly as T2T approaches are perfected. Overall, these studies will allow us to better understand the contributions of MEIs and their source elements to human diseases, both in the germline and in diverse somatic tissues.

## 8. SVA and *Alu* Source Elements

Like FL-L1Hs source elements, SVA and *Alu* elements also generate “offspring” insertions from source elements that are located throughout the human genome. SVA source elements can produce 5′ and 3′ transductions, which can be used to track new SVA offspring insertions to the source elements that produced them [47]. *Alu* elements, in contrast, generally do not produce flanking transductions (or they produce very short transductions on the order of a few base pairs). However, it may be possible to track source/offspring *Alu* relationships using sets of interior mutations, which often are uniquely found in specific *Alu* element copies [72]. As additional interior *Alu* sequences are fully discovered with long-read approaches, this may become increasingly possible on a broader scale. Some *Alu* elements only have the interior mutations that define the subfamily of the element (such as *Alu* Ya5, where five specific interior changes define the subfamily). Since there are thousands of element copies that fall into this category, these elements will be particularly challenging to track in terms of source/offspring relationships. However, as with FL-L1Hs and SVA source elements, it may be possible to study the regulation of at least some *Alu* source elements using interior mutation patterns that are unique to specific copies.

## 9. Conclusions

Historically, several methods have been used to discover new MEIs and study their impact on human genomes. We are now entering a new phase of MEI discovery that uses whole genome sequences and bioinformatics tools to discover such elements, and we expect that this approach will continue to grow rapidly as the costs of Illumina and long-read sequencing continue to drop. These tools also will allow us to discover and study the FL-L1Hs source elements that drive L1, *Alu*, and SVA retrotransposition. Overall, these WGS-based methods are expected to greatly expand our knowledge of MEI mutagenesis in humans and allow us to study the impact of these newly-inserted MEIs on human traits and diseases.

As we have increasingly moved to long-read assembled genomes, several new MEI discovery methods have been developed that use genome assemblies (and/or long-read mapping) to identify novel MEIs and to annotate them (e.g., PALMER and MEIGA [47]). In most cases, after the initial MEIs are discovered, approaches that have been developed for short-read data (or similar approaches) are used to fully annotate the target site duplications, subfamilies, and other features [12,14,47]. A major advantage of long-read assemblies is that the full interior sequences of the MEIs are recovered (whereas only the junction sequences are recovered with short-read approaches).

We expect that population-scale sequencing studies will continue to expand, which will enable the identification of an unprecedented number of MEIs that impact human genetics, diseases, and evolution (as we have seen with several dozen MEIs thus far that have disrupted genes in the germline [39,40,41,42,43,44] and somatic tissues [24,25,26,27,28,29,30,31,32]). Any MEI that disrupts a functionally important genomic segment can potentially impact human traits, diseases, and evolution. The new approaches outlined above will empower these studies by promoting MEI discovery in a much larger slice of humans with various traits and diseases. These studies also will allow us to study the impact of MEIs that have differentially impacted the world’s populations in terms of human traits, evolution, and health.
